# Chronic subdural haematoma during the COVID-19 lockdown period: late presentation with a longer interval from the initial head injury to the final presentation and diagnosis

**DOI:** 10.1186/s41016-020-00229-7

**Published:** 2021-01-08

**Authors:** David Yuen Chung Chan, Wai Sang Poon, Danny Tat Ming Chan, Wai Kit Mak, George Kwok Chu Wong

**Affiliations:** Division of Neurosurgery, Department of Surgery, Faculty of Medicine, The Chinese University of Hong Kong, Prince of Wales Hospital, Hong Kong SAR, China

**Keywords:** Chronic subdural haematoma, Novel coronavirus, COVID-19, Head injury

## Abstract

**Background:**

The COVID-19 novel coronavirus is contagious, and the mortality is higher in the elderly population. Lockdown in different parts of the world has been imposed since January 2020. Chronic subdural haematoma (cSDH) has a unique natural history in which symptoms can be non-specific, and the onset is insidious. This study aims to evaluate the impact of the COVID-19 pandemic on the presentation of cSDH.

**Methods:**

Consecutive adult cSDH patients admitted from 1 March 2020 to 30 April 2020 were reviewed. Exclusion criteria including those who had no definite history of head injury or the diagnosis of cSDH were made from a scheduled follow-up scan. Corresponding data during the same period in 2019 were reviewed for comparison. The primary outcome was the interval between the initial head injury and the final radiological diagnosis of cSDH. Secondary outcomes include Markwalder chronic subdural haematoma grade upon admission, length of stay in the acute hospital, and the modified Rankin scale (mRS) upon discharge.

**Results:**

For the primary outcome, the average interval between head injury and the diagnosis of cSDH was significantly longer at 56.6 days (49 to 74 days, SD 9.83 days) during the period from March to April 2020, versus 29.4 days (17 to 42 days, SD 8.59 days) in 2019 for the corresponding period (*p* = 0.00703). There was no significant difference in the functional outcome upon discharge.

**Conclusions:**

cSDH patients can present late during the COVID-19 lockdown period. The functional outcome was comparable when operations for drainage were timely performed.

## Background

The COVID-19 novel coronavirus is highly contagious, and the mortality is higher in the elderly population [1]. Lockdown in different parts of the world has been imposed since late January 2020 [2]. Non-essential workers including the general population were instructed to stay at home, and many had avoided attending the hospitals or clinics during the lockdown period. In the hospital, non-urgent operations were suspended [3]. The unique nature of chronic subdural hematoma (cSDH) in which the symptoms were non-specific and the onset was insidious had clinical implications during the lockdown. The aim of this study is to evaluate the impact of the COVID-19 pandemic on the presentation of cSDH.

## Methods

We conducted a retrospective review of consecutive patients admitted to the Division of Neurosurgery at Prince of Wales Hospital from 1 March 2020 to 30 April 2020. Inclusion criteria were all adult patients with (1) a diagnosis code of chronic subdural haematoma from the International Classification of Diseases ninth revision (ICD-9) “432.1(1)” and (2) a radiological diagnosis of cSDH as evidenced from either a computed tomographic (CT) scan or magnetic resonance imaging (MRI) of the brain. Exclusion criteria include those who had (1) no definite history of head injury, (2) those whose initial scan showing acute subdural haematoma (aSDH) or traumatic subarachnoid haemorrhage (tSAH) and cSDH diagnosed from an elective scheduled follow-up scan, or (3) cSDH from other secondary causes such as intracranial hypotension or other underlying haematological disorders. Corresponding data during the same period in the previous year from 1 March 2019 to 30 April 2019 were reviewed for comparison. The primary outcome was the interval between the initial head injury and the radiological diagnosis of cSDH. Secondary outcomes include Markwalder chronic subdural haematoma grade upon admission, length of stay in the acute hospital, and the modified Rankin scale (mRS) upon discharge.

Statistical analysis was performed with the chi-square test, Fisher’s exact test, and unpaired Student *t* test. Statistical analysis was performed with the Statistical Package for the Social Sciences for Microsoft Windows Version 24.0.0 (IBM SPSS Inc., Chicago, IL, USA).

## Results

In total, 171 patients with cSDH were admitted during the study period. The total number of cSDH patients admitted during the 2020 lockdown period were similar to the same period 1 year ago, in which 86 were admitted from 1 March 2020 to 30 April 2020 (the 2020 group) and 85 were admitted from 1 March 2019 to 30 April 2019 (the 2019 group). The age was comparable at 76.0 (44–102) years old for the 2020 group versus 78.2 (41–101) years old for the 2019 group. The majority of the cSDHs were male, which accounted for 73.26% (63/86) in the 2020 group versus 84.71% (72/85) in the 2019 group.

Sixty-eight cSDH patients in the 2020 group and 69 in the 2019 group were elective admissions for scheduled scans for the detection of any development of cSDH (from a previous aSDH or tSAH) or for monitoring any progression or recurrence of a previously thin cSDH. These groups of patients were excluded from the analysis according to the inclusion and exclusion criteria. For these elective admissions for scheduled scans for detection of any progression of cSDH or recurrence, 3 out of 68 (4.54%) of the cSDH patients in the 2020 group and 0 out of the 70 (0%) in the 2019 group required burr hole drainage.

Sixty-six cSDH patients in the 2020 group and 70 in the 2019 group were discharged from the hospital the same day after clinical assessment and undergoing computed tomographic (CT) scan the same day. 23.3% (20/86) cSDH patients in the 2020 group versus 17.7% (15/85) in the 2019 group required more than 1-day inpatient hospitalisation for the treatment of cSDH (*p* = 0.363).

20.9% (18/86) cSDH patients in the 2020 group versus 18.8% (16/85) in the 2019 group were emergency admissions (*p* = 0.730). These patients were included for analysis as they did not fulfill the exclusion criteria. For the emergency admissions, 94.4% (17/18) of cSDH patients in the 2020 group versus 93.8% (15/16) in the 2019 group required burr hole drainage during the same admission episode (*p* = 0.816).

For the detailed analysis of 18 symptomatic cSDH patients in the 2020 group who required burr hole drainage, eight had no history of head injury, one was intracranial hypotension, and nine had a history of head injury, in which five had an interval preceding head injury with attendance to A&E and CT head performed. For the 2019 group with 16 symptomatic cSDH, seven had no history of head injury and nine had a history of head injury, in which six had an interval preceding episode of head injury.

The average time between head injury and the diagnosis of cSDH was 29.4 days (17 to 42 days, SD 8.59 days) in the 2019 group for the six patients who had a clear history of a preceding head injury, versus 56.6 days (49 to 74 days, SD 9.83 days) for March to April 2020 with five patients, which was significantly longer (*p* = 0.00703).

For the Markwalder grade upon admission, there was no significant difference between the two groups. The median Markwalder grade at admission was 1 for both groups. For the 2020 group, 75% of the admission Markwalder grade was 1 and 25% had an admission Markwalder grade of 2. For the 2019 group, 70% had an admission Markwalder grade of 1 and 30% had a Markwalder grade of 2 (*p* = 0.354).

The overall length of stay in the acute hospital was 8.9 days (4 to 19 days) in the 2020 group versus 9.2 days (2 to 25 days) in the 2019 group (*p* = 0.425).

The functional outcomes in terms of mRS upon discharge were comparable, in which 60% (12/20) in the 2020 group had good outcomes (mRS 0–3), versus 53.3% (8/15) in the 2019 group (*p* = 0.694).

## Discussion

**Fig. 1:**
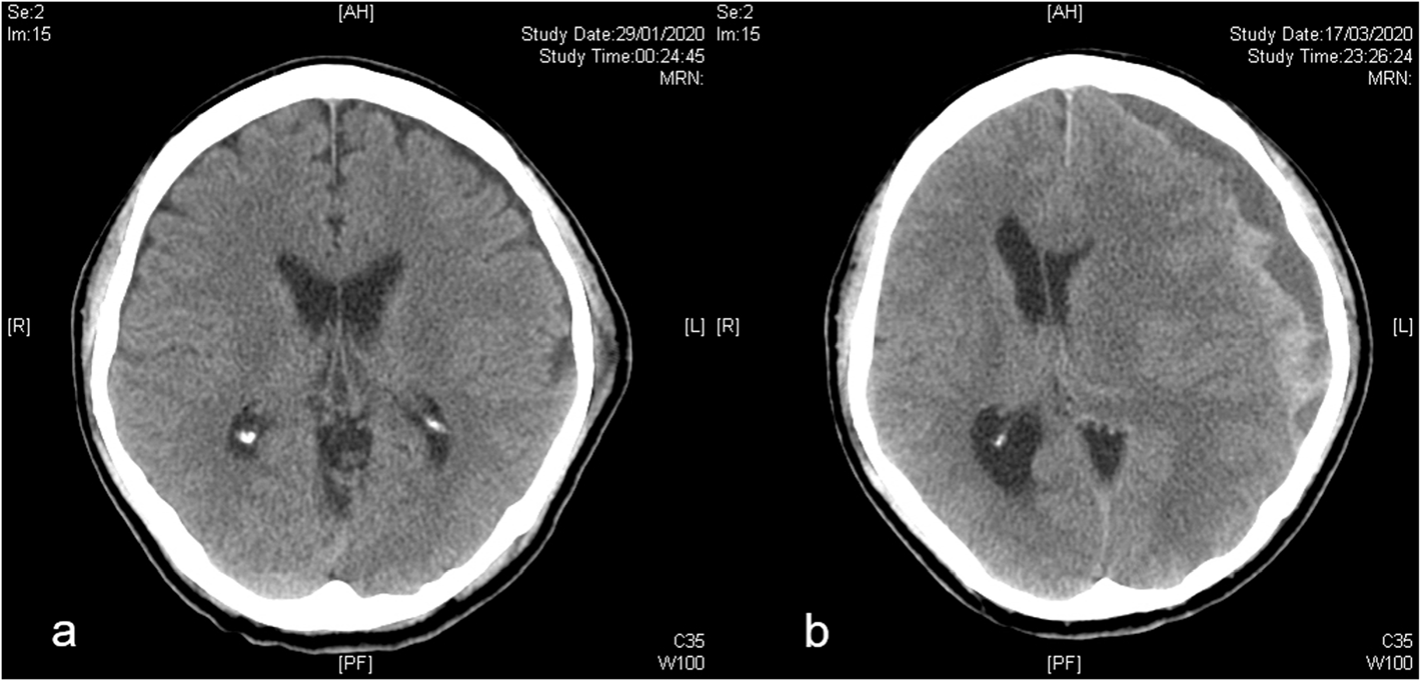
**a** Computed tomographic (CT) scan of the brain of a 59-year-old gentleman at the time of injury. He had a minor head injury with retrograde amnesia. CT scan showed a left-sided scalp hematoma but no intracranial hemorrhage and he was discharged with medical advice. Subsequently, he had a progressively worsening headache. **b** He finally re-attended the hospital 7 weeks after the initial minor head injury. The new CT showed a left-sided chronic subdural hematoma with mass effect and midline shift. He had a good recovery after emergency burr hole drainage

**Fig. 2:**
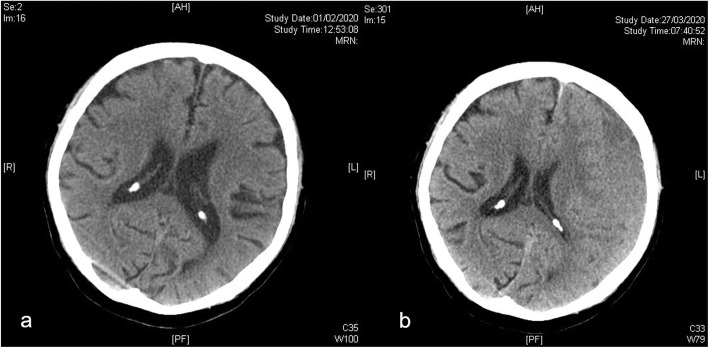
**a** CT brain scan of an 86-year-old gentleman at the time of injury. He had a minor head injury due to postural hypotension and his anti-hypertensive medication was titrated. CT scan initially showed no intracranial hemorrhage and he was discharged with medical advice. He subsequently had a gradual onset of worsening dizziness. **b** He presented to the hospital 7 weeks and 5 days after his initial minor head injury. The new CT showed a newly diagnosed left-sided chronic subdural hematoma with mass effect and midline shift. He had a good recovery after emergency burr hole drainage

**Fig. 3:**
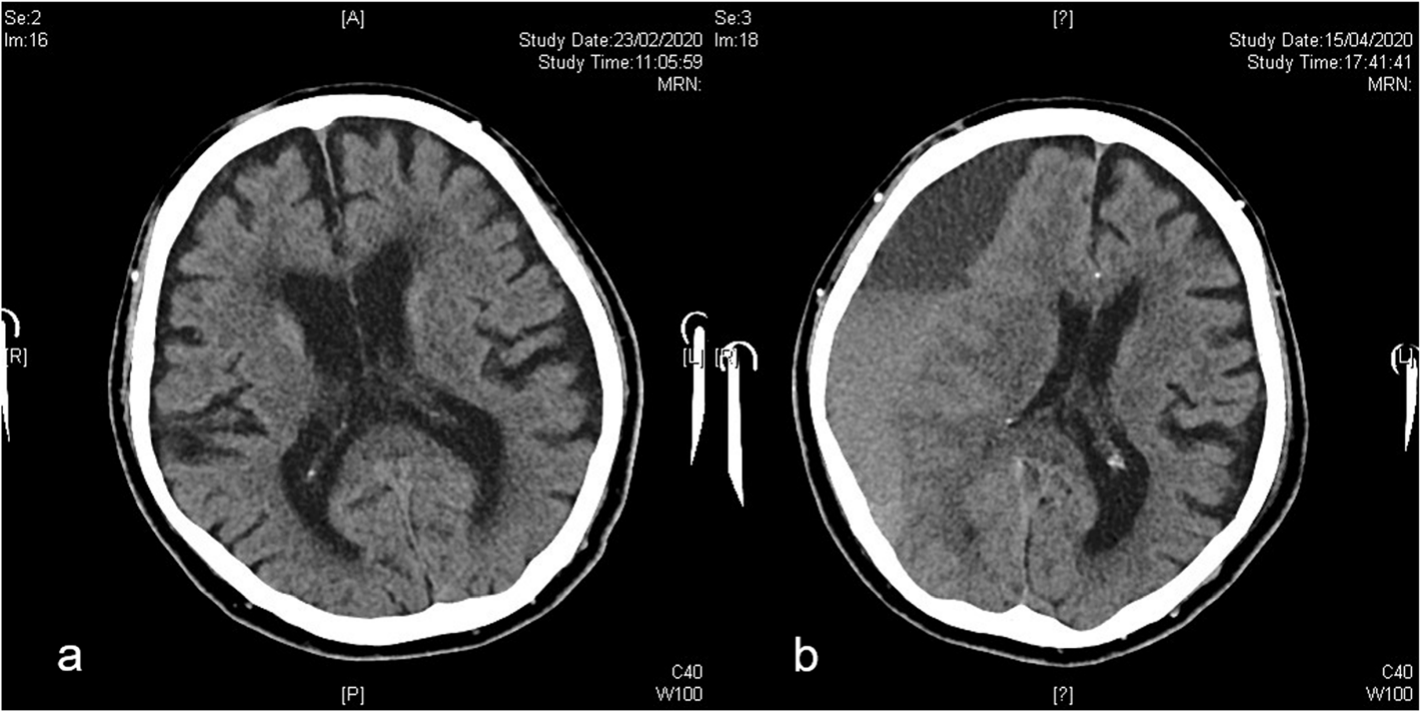
**a** CT brain scan of a 97-year-old gentleman at the time of injury. He had slipped and had a fall on level ground with a minor head injury. CT scan initially showed no intracranial hemorrhage and he was discharged with medical advice. Subsequently, he had progressively worsening left-sided weakness and clumsiness but did not go to the hospital immediately in view of the pandemic. **b** He attended the hospital 7 weeks and 3 days after his initial minor head injury. The new CT showed a newly diagnosed right-sided chronic subdural hematoma with mass effect and midline shift. Despite the delay in presentation, he had a good recovery after emergency burr hole drainage

Since March 2020, we have observed a pattern of delay in the presentation of chronic subdural haematoma patients after their initial head injury during this period of lockdown (Figs. 1, 2 and 3). The figures illustrated patients who had minor head injuries in January or February 2020. Their initial CT scan of the brain at the time of injury showed no major intracranial haemorrhage (Figs. 1, 2 and 3a). They went home with medical advice. Subsequently, they gradually deteriorated with non-specific symptoms including unsteady gait, dizziness, or clumsiness. They initially avoided attending the hospital or clinic due to the COVID-19 pandemic. Finally, at around 7 to 10 weeks after their initial minor head injury (Figs. 1, 2 and 3b), they were diagnosed with cSDH. In our tertiary neurosurgical centre, this delay pattern was not observed in other major neurosurgical conditions including ruptured aneurysms, stroke, or spinal cord injury during this lockdown period.

cSDH can be associated with minor head injuries [4]. In those who can recall a history of head injury, the timing of the injury was usually around 4 weeks before the CSDH was developed and diagnosed and it ranges from 2 to 6 weeks [5–9]. The time frame can even be shorter for those who were on antiplatelets or anticoagulants [10–12]. Panciani et al. had reported a worse surgical outcome of cSDH patients during this COVID-19 pandemic [13]. In their series of COVID-19-positive cSDH patients, 4 out of 5 passed away after the operations. The specific interval between the initial head injury and the diagnosis of cSDH was not reported in their study. For our 2020 cohort, there was no mortality within 30 days. Although a late presentation during this lockdown period was observed, the overall outcome in terms of mRS was comparable. This signifies good functional outcome can be achieved when operations were timely performed for drainage [14].

Some of the cSDH patients might have visited other hospitals or other neurosurgical centre when they had a minor head injury previously. They then visit our centre as an emergency due to deterioration. All these patients had been included for analysis in our study. The strength of our study is the precise inclusion and exclusion criteria so that we are studying a specifically defined group of cSDH. The weakness of our study is the small sample size. However, the difference is large and a significant outcome was observed even with small sample size. Further studies can be performed in other parts of the world to assess if there is a similar delay pattern of presentation.

The majority of cSDH were elderly, and many of them cannot recall the exact date of symptoms onset. Some geriatric patients recalled vague answers such as a few days of headache but their family members said the patients were slow with unsteady gait for a week or two already but did not attend the hospital due to COVID-19. Data in our current study based on head injury date and the date of radiological diagnosis were objective and accurate. It is an objective way to evaluate the clinical situation during this COVID-19 era.

Contributing factors for late presentation during the COVID-19 period include the distinct natural history of cSDH in which the symptoms can be non-specific and the onset was insidious [15]. Other possibilities include less-frequent visits from social workers or other family members during the lockdown period. We would like to raise awareness for this situation, and clinicians should remain vigilant for this insidious and curable neurosurgical condition during this pandemic.

## Conclusions

Chronic subdural hematoma patients presented late during the COVID-19 lockdown period with a longer interval from the initial head injury to the time of reaching the final diagnosis.

## Data Availability

All data generated or analysed during this study are included in this published article.

## Abbreviations

aSDH: Acute subdural haematoma
COVID-19: Coronavirus disease 2019
cSDH: Chronic subdural haematoma
CT: Computed tomographic scan
ICD-9: International Classification of Diseases ninth revision
MRI: Magnetic resonance imaging
mRS: Modified Rankin scale
SPSS: Statistical Package for the Social Sciences for Microsoft Windows
SD: Standard deviation
tSAH: Traumatic subarachnoid haemorrhage

## References

[1] Chen T, Wu D, Chen H, Yan W, Yang D, Chen G (2020). Clinical characteristics of 113 deceased patients with coronavirus disease 2019: retrospective study. BMJ.

[2] Leung K, Wu JT, Liu D, Leung GM (2020). First-wave COVID-19 transmissibility and severity in China outside Hubei after control measures, and second-wave scenario planning: a modelling impact assessment. Lancet..

[3] Chan DYC, Chan DTM, Mak WK, Wong GKC, Poon WS (2020). Letter: Rongeurs, Neurosurgeons, and COVID-19: how do we protect health care personnel during neurosurgical operations in the midst of aerosol-generation from high-speed drills?. Neurosurgery.

[4] Santarius T, Kirkpatrick PJ, Ganesan D, Chia HL, Jalloh I, Smielewski P (2009). Use of drains versus no drains after burr-hole evacuation of chronic subdural haematoma: a randomised controlled trial. Lancet.

[5] Edlmann E, Holl DC, Lingsma HF, Bartek J, Bartley A, Duerinck J (2020). Systematic review of current randomised control trials in chronic subdural haematoma and proposal for an international collaborative approach. Acta Neurochir.

[6] Kolias AG, Chari A, Santarius T, Hutchinson PJ (2014). Chronic subdural haematoma: modern management and emerging therapies. Nat Rev Neurol.

[7] Stanisic M, Lyngstadaas SP, Pripp AH, Aasen AO, Lindegaard KF, Ivanovic J (2012). Chemokines as markers of local inflammation and angiogenesis in patients with chronic subdural hematoma: a prospective study. Acta Neurochir.

[8] Chan DYC, Woo PYM, Poon WS (2014). Chronic subdural hematoma: to drain or not to drain? This is the question. World Neurosurg.

[9] Chan DYC, Sun TFD, Poon WS (2015). Steroid for chronic subdural hematoma? A prospective phase IIB pilot randomized controlled trial on the use of dexamethasone with surgical drainage for the reduction of recurrence with reoperation. Chinese Neurosurg J.

[10] Tailor J, Fernando D, Sidhu Z, Foley R, Abeysinghe KD, Walsh DC (2017). Clinical audit effectively bridges the evidence-practice gap in chronic subdural haematoma management. Acta Neurochir.

[11] Chan DYC, Chan DTM, Sun TFD, Ng SCP, Wong GKC, Poon WS (2017). The use of atorvastatin for chronic subdural haematoma: a retrospective cohort comparison study. Br J Neurosurg.

[12] Chari A, Clemente Morgado T, Rigamonti D (2014). Recommencement of anticoagulation in chronic subdural haematoma: a systematic review and meta-analysis. Br J Neurosurg.

[13] Panciani PP, Saraceno G, Zanin L, Renisi G, Signorini L, Fontanella MM. Letter: COVID-19 infection affects surgical outcome of chronic subdural hematoma. Neurosurgery. 2020;87(2):E167–71. 10.1093/neuros/nyaa140 PMC7188118 32304213

[14] Chan DYC, Woo PYM, Mak CHK, Chu ACH, Li CCH, Ko NMW (2017). Use of subdural drain for chronic subdural haematoma? A 4-year multi-centre observational study of 302 cases. J Clin Neurosci.

[15] Kolias AG, Hutchinson PJ, Santarius T (2017). Chronic subdural haematoma: disseminating and implementing best practice. Acta Neurochir.

